# Complete mitochondrial genome of natural diploid loaches *Misgurnus anguillicaudatus* from the Taihu Lake

**DOI:** 10.1080/23802359.2018.1467227

**Published:** 2018-05-11

**Authors:** Guosong Zhang, Daoyu Zhu, Xiaodong Li, Xia Liang, Kejun Cai, Haili Zhang, Guisheng Zhang

**Affiliations:** a School of Agriculture and Bioengineering, Heze University, Heze, Shandong, China;; b Chemistry and Bioengineering School, Hechi University, Yizhou, Guangxi, China;; c Department of Life Science, Huzhou University, Huzhou, Zhejiang, China

**Keywords:** Taihu loach, *Misgurnus*, mitogenome, conservation

## Abstract

*Misgurnus anguillicaudatus* is a highly valued, aquaculture-relevant food fish in East Asian countries. Because of overfishing and environmental pollution, the number of wild Taihu loach has been sharply decreased in these years. In this study, the complete mitochondrial genome of Taihu loach was obtained by PCR. The genome is 16,646 bp in length, including 2 ribosomal RNA genes. Thirteen proteins-coding genes, 22 transfer RNA genes, and a non-coding control region, the gene composition and order of which was similar to most reported from other vertebrates. Sequence analysis showed that the overall base composition is 29.8% for A, 28.2% for T, 25.7% for C, and 16.3% for G. The phylogenetic tree showed that Misgurnus family got together for one branch, which includes Taihu *M. anguillicaudatus*, and the other loaches had their own branches. Also the mitochondrial genome sequence of *M. anguillicaudatus* were aligned by BLAST, compared with Cobitinae the sequence similarity could reach >94%, and the similarity to Misgurnus was >99%.

Cyprinid loach, *Misgurnus anguillicaudatus* (Cypriniformes; Cobitidae), a small-sized freshwater fish species, is a highly valued, aquaculture-relevant food fish in East Asian countries. This loach species has also been given much attention as an important model organism to study developmental biology, polyploidy evolution, and genetic breeding (Zhang et al. 2014; Liu et al. 2016). Because of overfishing and environmental pollution of Taihu Lake, the number of natural wild *M. anguillicaudatus* has been sharply decreased in these years. Therefore, it is very important to characterize the complete mitogenome of this species, which could be a fundamental basis to address genetic identity and diversity in future conservation program of this rarely occurring (Sang et al. 2017).

In this study, we sequenced the complete mitogenome of *M. anguillicaudatus* with a GenBank accession number MG938590. The voucher specimen was collected from Zhili Fanyi aquaculture base, north latitude 30°22″ and east longitude 120°25″, Huzhou city, China. They were preserved in 95% alcohol, which were stored in biology herbarium of Heze University. All the DNA were extracted using phenol–chloroform extraction methods and stored at −80 °C. The mitogenome were amplified by primers which were initially published (Zeng et al. 2012). The entire mitogenome sequence of Taihu loach was 16,646 bp in length, consisting of 13 protein-coding genes (PCGs), 2 ribosomal RNA (rRNA) genes, 22 transfer RNA (tRNA) genes, one replication origin (OL) and one control region (D-loop). From the base composition analysis, the percent A + T content was 58.0% (29.8% for A, 28.2% for T, 25.7% for C, and 16.3% for G). Twelve PCGs, 14 tRNA genes and two rRNA genes were located on the heavy strand (H-strand), while one PCG (ND6) and eight tRNA genes (tRNA^Gln^, tRNA^Ala^, tRNA^Asn^, tRNA^Cys^, tRNA^Tyr^, tRNA^Ser^, tRNA^Glu^, and tRNA^Pro^) on the light strand (L strand). Eight PCGs (ND1, COI, COII, ATP8, ATP6, ND4L, ND5 and ND6) were terminated with TAA stop codon and three PCGs (ND2, ND3 and ND4) ended with TAG. On the other hand, remaining two PCGs (COIII and CYTB) ended with the incomplete stop codon represented as a single T. Frame overlapping occurred at three pairs of PCGs. ATP8 and ATP6 overlapped by 10 nucleotides (nt), ND4L and ND4 by 7 nt, and ND5 and ND6 (encoded on opposing stand) by 4 nt. Two ribosomal RNA genes, 12S rRNA (953 bp) and 16S rRNA (1679 bp) were located between tRNA^Phe^ and tRNA^Leu^ with a separation by the tRNA^Val^ as seen in other vertebrate mitogenomes.

To determine the taxonomic status of *M. anguillicaudatus*, we performed the phylogenetic relationship of Taihu *M. anguillicaudatus* stock with other natural populations in loach as inferred by entire mitogenome (Dai et al. 2016). The phylogenetic tree showed that Misgurnus family got together for one branch, which includes Taihu *M. anguillicaudatus*, and the other loaches had their own branches (Figure 1). Also the mitochondrial genome sequence of *M. anguillicaudatus* were aligned by BLAST, compared with Cobitinae the sequence similarity could reach >94%, and the similarity to Misgurnus was >99%.

**Figure 1.**
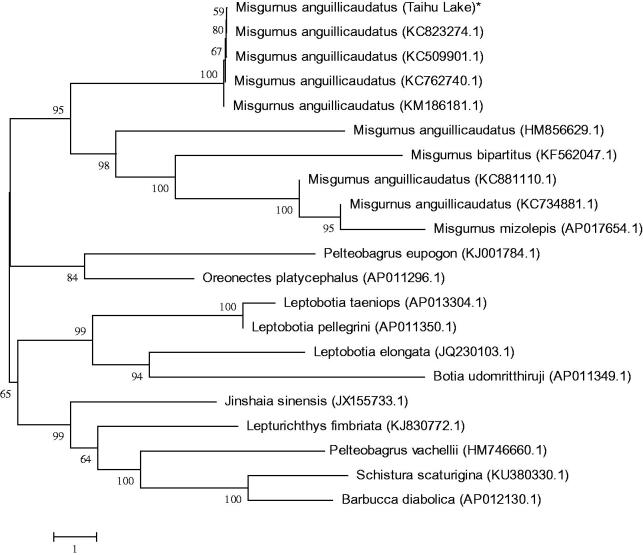
Phylogenetic relationship of Taihu *M. anguillicaudatus* stock with other loach as inferred by entire mitogenome. *The Taihu loach (accession number: MG938590) in the position of the evolutionary tree. Trees were reconstructed using MEGA 7 program (Kumar, Tamura, Nei) with neighbour-joining method. Numbers above branches are bootstrap values by 1000 replicates. The phylogenetic tree showed that Taihu loach to be one of *Misgurnus*, and the other loaches had their own branches.
